# Two Feet-One Hand Syndrome

**DOI:** 10.7759/cureus.20758

**Published:** 2021-12-27

**Authors:** Junki Mizumoto

**Affiliations:** 1 Department of Medical Education Studies, International Research Center for Medical Education, Graduate School of Medicine, University of Tokyo, Tokyo, JPN

**Keywords:** two feet-one hand syndrome, tinea pedis, tinea manuum, ringworm, geriatrics, dermatology

## Abstract

Ringworm infection is a common but frequently misdiagnosed skin disease. An 81-year-old woman presented with a complaint of mild itch of the third and fourth fingers of her right hand and the toes of both feet. A crusted rash was seen on the right hand and both feet. The results of potassium hydroxide testing were positive for filamentous fungi. The diagnosis of two feet-one hand syndrome was made. The rash was treated successfully by topical ketoconazole. Recognition of this typical distribution of the rash may help make a prompt diagnosis of ringworm infection.

## Introduction

Ringworm is one of the most common agents in skin infection. Its diagnosis is often difficult because of many mimics [1]. Ringworm infection may cause not only somatic symptoms such as itch but also embarrassment [2]. Ringworm infection sometimes involves patients' unilateral hand (tinea manuum) and bilateral feet (tinea pedis), called as two feet-one hand syndrome. It is reported that more than 80% of patients with tinea manuum also have tinea pedis [3]. This characteristic distribution helps physicians make a correct diagnosis. Herein, we reported a case of two feet-one hand syndrome via a thorough routine checkup. The patient did not report her skin disturbance because of embarrassment.

## Case presentation

An 81-year-old woman presented to a long-term nursing care home and began to see a home-visit doctor regularly. The patient had lived alone in her house until two months before, when a neighbor reported to a community public health center that she was shabbily clothed and her house was in a mess. Public health nurses found that her activities of daily living were limited because of cognitive decline. The patient was admitted to a hospital as an emergency evacuation. No health problem except mild dementia was pointed out, and she was discharged from the nursing care home.

**Figure 1:**
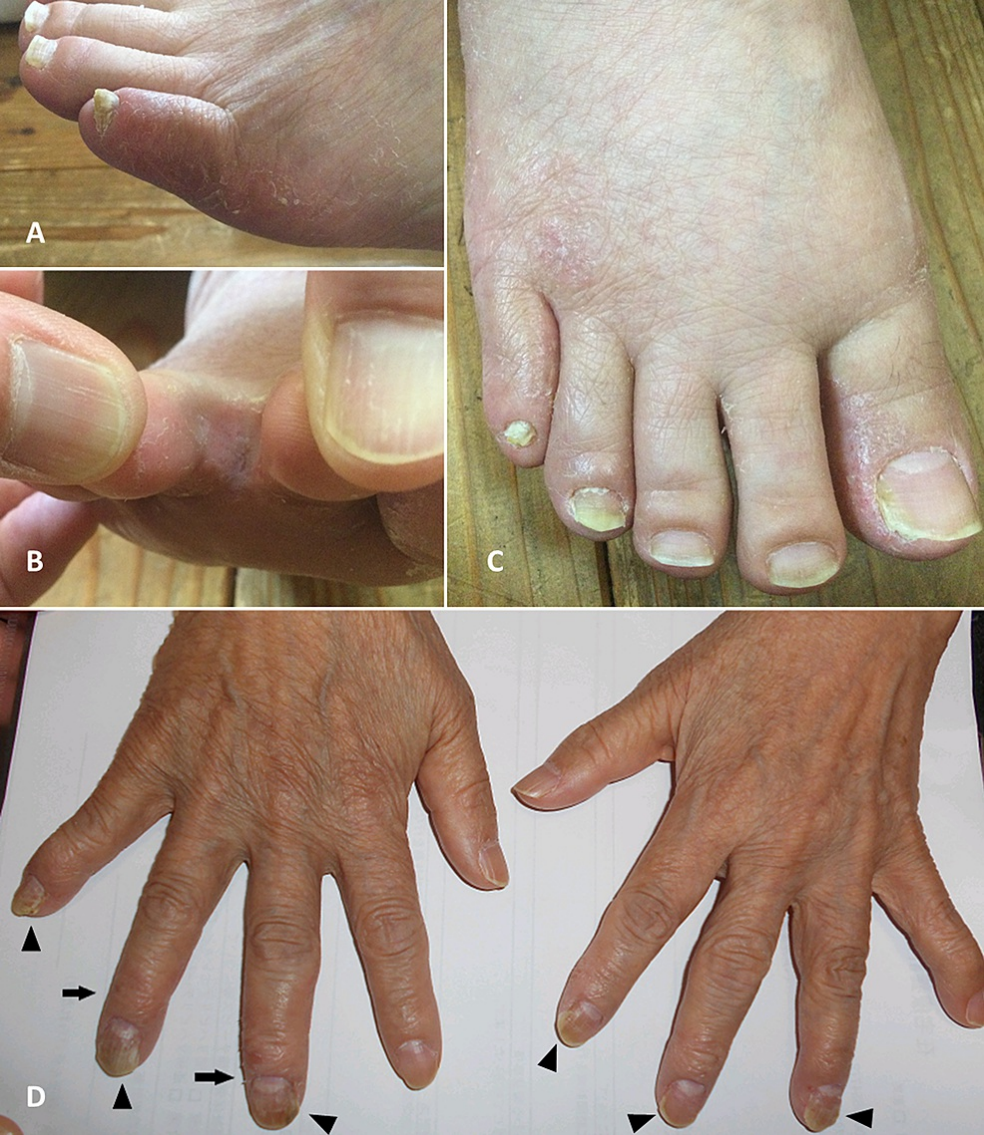
The patient's hands and feet (A) Tinea pedis on the fifth toe of the left foot. (B) Tinea pedis between the first and second toes of the left foot. (C) Periungual tinea pedis on the first, fourth, and fifth toes of the right foot. (D) Periungual tinea manuum on the third and fourth fingers of the right hand; onychomycosis on the third, fourth, and fifth nails of the right hand, and on the second, third, and fourth nails of the left hand.

At the first encounter of the patient and a home-visit doctor, a routine physical examination was performed. A crusted rash was seen on both feet (Figures 1A, 1B), especially in the interdigital areas (Figure 1C). The third and fourth fingers of the right hand had an itchy rash, and thickened nails were seen on the third, fourth, and fifth fingers of the right hand (Figure 1D). There was no swelling or heat indicating inflammation. When the patient was asked about these skin changes, she complained of mild itch of the third and fourth fingers of her right hand and the toes of both feet. She also said that she did not tell the symptoms because of embarrassment. A review of information from the hospital revealed no relevant background diseases including diabetes mellitus or immunocompromised conditions.

According to the typical distribution, ringworm infection (two feet-one hand syndrome) was suspected. The results of potassium hydroxide (KOH) testing of crusted skin scrapings were positive for filamentous fungi. The diagnosis of tinea pedis with tinea manus was made. Topical ketoconazole was administered, and the rash improved in the next two months.

## Discussion

Bilateral tinea pedis with coexistent unilateral tinea manuum is called two feet-one hand syndrome [4]. Physicians can suspect ringworm infection when seeing this typical rash distribution. This syndrome is highly associated with onychomycosis, as in this case, and around 6% of patients with onychomycosis developed two feet-one hand syndrome [5]. Typically, tinea pedis occurs at an earlier age than tinea manuum [6]. Ringworm is thought to be transmitted from one foot to the hand by excoriating the soles of the feet and picking toenails and is then transmitted from the hand to the other foot [3,6,7]. In some cases, tinea manuum develops in both hands, contrary to the name “one hand” [8]. The prevalence of “two feet-two hand syndrome” remains unknown.

A topical antifungal agent is usually selected for treating this syndrome. However, these agents sometimes cause contact dermatitis, which is difficult to diagnose only by visual inspection [9]. Other differential diagnoses include psoriasis, chronic eczema, and hand-foot syndrome [10-12]. Two feet-one hand syndrome is sometimes relapsing [13], and inappropriate treatment such as topical steroid under incorrect diagnosis may worsen the condition. KOH testing is crucial for the correct diagnosis [1].

Physicians should know the typical distribution of two feet-one hand syndrome because this recognition may help make a diagnosis even in a setting lacking in medical resources such as a home-visiting care. Examination of the feet is often overlooked partly because of spending too short time on physical examination and physicians’ laziness [14]. This case highlights the importance of thorough physical examination in a routine manner.

## Conclusions

A crusted rash distributed in two feet and one hand may suggest ringworm infection. Physicians should perform KOH testing in seeing this typical distribution to make a correct and immediate diagnosis because ringworm infection has many mimics. Topical antifungal agent is the first choice of therapy. Patients sometimes do not report skin changes by themselves because of embarrassment. Physicians often skip examination of the feet. A thorough physical examination is needed, especially at the first encounter of older patients.
